# A CREATIVE approach to designing a contemporary medical curriculum

**DOI:** 10.15694/mep.2020.000038.1

**Published:** 2020-03-06

**Authors:** Shazia Iqbal, Shahzad Ahmad, David Taylor, Jaye McIsaac, Turky H. Almigbal

**Affiliations:** 1 Alfarabi College of Medicine Riyadh; 2 Fatima Memorial Medical and Dental College Lahore Pakistan; 3 Gulf Medical University Ajman UAE; 4 Auckland University of Technology New Zealand; 5 King Saud University

**Keywords:** Outcome-based Education (OBE), SPICES based integrated curriculum, contemporary medical curriculum, Medical education, curriculum evaluation, OBE Integrated curriculum review and reforms

## Abstract

This article was migrated. The article was marked as recommended.

In medical education, Outcome-based Education (OBE) is facing many challenges due to the rapid proliferation in biomedical sciences; technology-enhanced learning; globalization of health system, and the availability of various pedagogical techniques. A steady stream of evidence-based developments challenges those who are responsible for sustaining and improving their medical curricula. Curriculum reform is a dynamic process and there is no explicit approach that can serve as a guide for a curriculum reviewer while making amendments in the OBE integrated curriculum. This study took place in a medical college in Saudi Arabia and it highlights the key components to be considered while reviewing the Bachelor in Medicine and Surgery (MBBS) course specifications and curriculum. It suggests the importance of course reports as the main driving force for curriculum reforms. Delineating major and minor changes in the curriculum and frequency of amendments in course specifications also remains a questionable task and requires established institutional guidelines. Furthermore, based on practical experience in the periodic curriculum review process, the authors suggest some best practice. We present a **“CREATIVE”** way forward which we have found invaluable, both for curriculum developers and for engaging with our stakeholders. This mnemonic spells out the key factors to consider while revisiting the curriculum from various angles. These factors are: **C**ourse **R**eport recommendations, **E**valuation tools (internal and external), **A**lignment of pedagogical strategies with assessment, **T**echnology, **I**nnovation, **V**ariations in the learning environment, and **E**stablishment of institutional guidelines. Application of these fundamental elements allowed us the development of a curriculum that meets the standards for international accreditation and helped the institution to form a cohesive team of educationalists.

## Introduction

In the current era, Outcome-based Education (OBE) faces many challenges due to the rapid proliferation in biomedical sciences; technology-enhanced learning (TEL); globalization of the health system, and the availability of various pedagogical techniques. In medical education, a steady stream of evidence-based developments challenges those who are responsible for sustaining and improving their medical curricula.

The change in the educational environment demands training of medical graduates in the curriculum, which is more enriched to instil the spirit of teamwork, collaboration and value communication skills and professionalism during patient care. There is also a demand to create an educational environment which is friendly, relaxed and that guides the physicians to overcome the stressful events in complex situations by promoting the self-awareness ( Elder *et al.*, 2007).

### The Problem

A wide range of literature argues for the demand for curriculum reorganisation and claims that curriculum reform is a dynamic process ( Cate, O. ten, 2007; Snelgrove *et al.*, 2009) However, there is a lack of exploration of the evaluation processes and guidelines on the review of the curriculum. There is no explicit approach, which can serve as a guide for curriculum reviewers while making amendments in the OBE integrated curriculum. Moreover, we have chosen the SPICES framework to help our thinking about the key implications for medical curriculum development in the context of Outcomes-based Education. This term is abbreviated for; student-centred, problem-based, integrated, community-based, elective and systematic approach ( Harden, 2000).

There must be evaluation tools and policies at institutional levels, which guide the reviewers, faculty members, decision-makers and stakeholders to adopt these changes thereby progressing transformation in the educational environment effectively ( Harden, 2002). There is an intense demand to regularly revisit the curriculum with multifocal lenses. Besides, there is a requirement to craft an approach to revise the OBE model in medical education undergraduate’s curriculum in order to ensure the ownership and decrease resistance to change in the curriculum (Elizondo‐Montemayor *et al.*, 2008).

Furthermore, the medical educators are struggling to implement, stabilise, and sustain OBE, however, the extent to which the planned outcome-based curriculum has been applied successfully needs a robust evaluation method ( Changiz, T. and Yousefy, A., 2006). There is a prerequisite of a standard model or paramount gauge to ensure the application of outcome-based education ( Harden, 2007b; van den Berg, 2004). In addition, there are specific requirements to evaluate the pedagogical methods enhanced by technology in order to establish the sustainability of TEL in medical education ( Cook and Ellaway, 2015).

In addition to the educational imperatives above, a range of treatment modalities amalgamated with advanced technologies, for example, robotic surgery, artificial intelligence and telepresence enforce modification of the curriculum content, employment and assessment plans ( Harden, 2007a) Finally, social accountability and the psychological, societal & cultural diversity in the practising community impose the curricular reforms ( Cuff and Forstag, 2019; Quintero, 2014).

**Figure 1.**
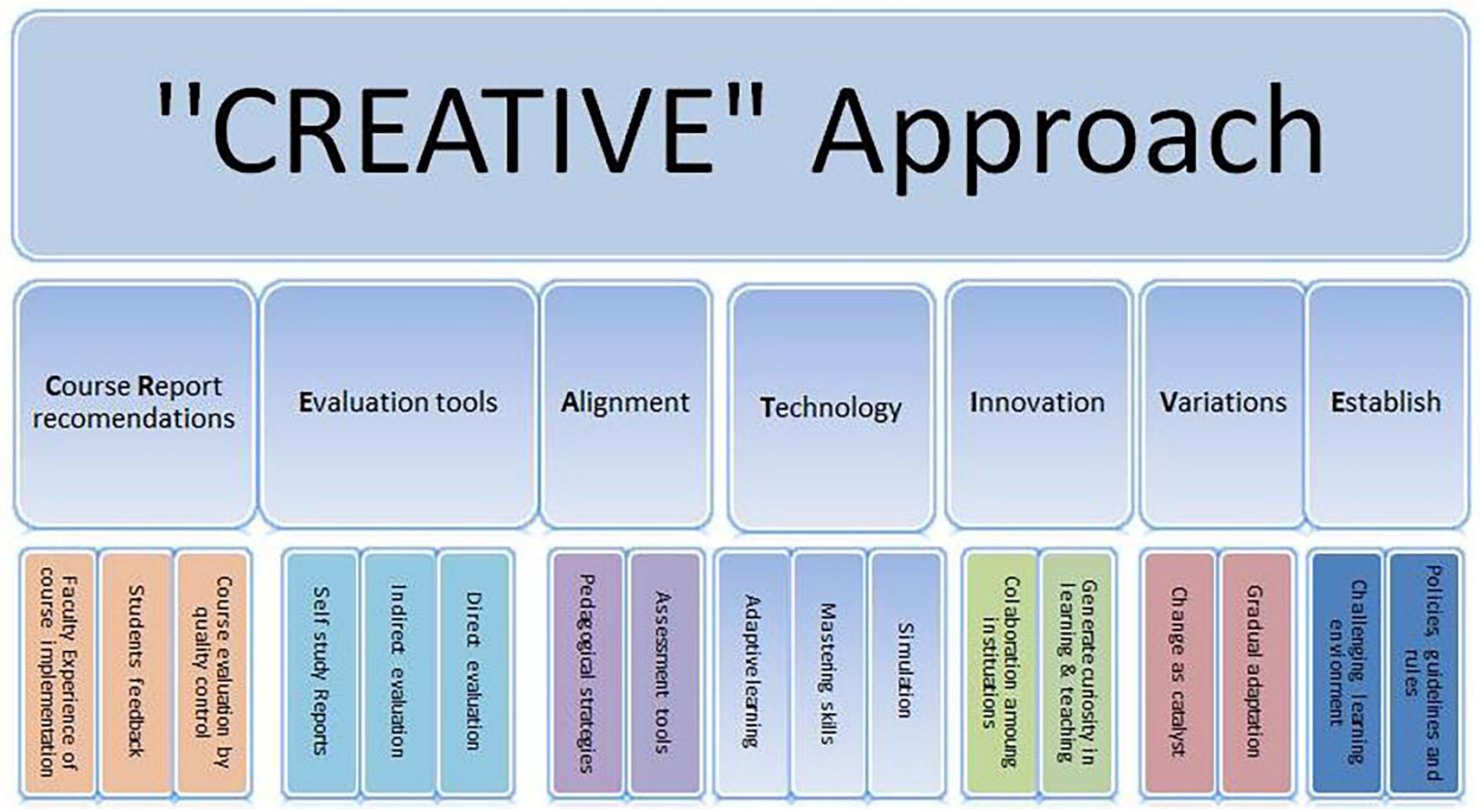
Implementation inventory suggests to follow “CREATIVE Approach” OBE integrated curriculum

This article addresses the practice of OBE integrated curriculum of medical instruction, at a medical college in Saudi Arabia. It highlights the key components to be considered while reviewing the Medicine and Surgery (MBBS) degree specifications and curriculum as a whole. It discusses the driving forces for curriculum manipulation and pinpoints the leading influences for curriculum management. It highlights the impact and consequences of decisions taken in view of recommendations made by stakeholders of the curriculum. Moreover, based on practical experience in the periodic curriculum review process, the authors suggest best practice points. In this article, we present a “CREATIVE” way forward which we have found invaluable, both for curriculum developers and for engaging with our stakeholders ( Figure 1). The CREATIVE mnemonic spells out the key factors to consider while revisiting the OBE and guides the reviewers to look into the curriculum from various angles. These factors are: **C**ourse **R**eport recommendations, **E**valuation tools (internal and external), **A**lignment of pedagogical strategies with assessment, **T**echnology, **I**nnovation, **V**ariations in the learning environment, and **E**stablishment of institutional guidelines. Application of these fundamental elements allowed us the development of a curriculum that meets the standards for international accreditation and helped the institution to form a cohesive team of educationalists. Moreover, it can be applicable for other professional OBE curriculum as well as the medical profession.

## Methods

### An intuitive approach to Outcome-based Education (OBE)

Alfarabi College of Medicine in Saudi Arabia offers a Bachelor in Medicine and Surgery (MBBS) programme of 212 credits, full time delivered over six years. We follow a competency/outcome-based curriculum built on the SPICES model ( Harden, 2007a). Here, specific learning outcomes provided by the Saudi Medical Education Directives (SaudiMED), act as a driver for curriculum planning ( Ali *et al.*, 2013; Awan *et al.*, 2018). SaudiMED framework of competency-based medical education is applied to most of the medical schools of Saudi Arabia. The stakeholders of SaudiMED work in coordination with the National Commission for Academic Assessment and Accreditation (NCAAA) and serve as a national benchmark for MBBS curriculum at Saudi Arabia. This model addresses the needs of society to produce competent medical practitioners with knowledge, attitudes, skills and ability to apply them in real-world practice (Snell & Frank 2010). The framework is considered similar to the CanMEDS framework; established by the Canadian Royal College of Physicians and Surgeons in 1996 ( Shadid *et al.*, 2019). CanMEDS is a framework that describes the abilities physicians require to effectively meet the health care needs of the people they serve. These abilities are grouped under the following seven roles: medical expert, collaborator, communicator, health advocate, leader, scholar, & professional.

A set of educational theories based on evidence underpin our curriculum. These theories include the learner-centred approach, self-directed learning, and theories of professional practice (teamwork, ethics, effective communication) ( Kaufman, 2003; Lindgren *et al.*, 2011). In addition to this, inspiration has been derived from reflective practice, constructivism, and professionalism ( Frank *et al,* 2010).

Our vision is to be a leading medical school in the Middle East region with dedicated continuing education development and to be internationally renowned for high-quality education. Our institution is keen to utilize well-qualified staff that provides significant and high-quality medical education using top-class, modern facilities.

### Curricular Design

The MBBS curriculum is characterised by integration, both horizontal and vertical throughout the programme. Basic medical sciences run from the beginning of the program in the form of system-based integration until the end of year three ( Brauer and Ferguson, 2015). Horizontal integration involves the division of basic sciences into various organ systems of the body. Vertical integration involves an early introduction to clinical skills, professionalism, ethics, evidence-based medicine and research through problem-based learning; seminars which focus on real-life clinical scenarios; community interaction and clinical placements. We employ the Z-model of curriculum integration where clinical sciences are considered alongside the basic sciences early in the curriculum and increase in relative importance. We have developed the curriculum according to the SPICES framework to make learning activities more student-centred, problem-based, integrated, community-based, elective-based and structured ( Harden, Sowden and Dunn, 1984).

## Results/Analysis

The OBE integrated curriculum planning and implementation need review process periodically. This study mentions the best practice points and recommendations on the basis of a detailed review of the curriculum over a period of four years (2015-2019) during eight cycles of OBE integrated curriculum analysis. The corresponding author’s key position and involvement in the curriculum review cycle; being an expert curriculum developer and chair of the Curriculum Management Committee (CMC) ascertain the employment of these recommendations in the OBE integrated curriculum. Most of the decisions within the course specifications about content mapping, applied pedagogical methods and schedules are devolved to course directors and content experts, who specify the appropriate pathways for reviewers to institute the final plans for curriculum.

## Discussion

### Course Report Recommendations

**Figure 2.**
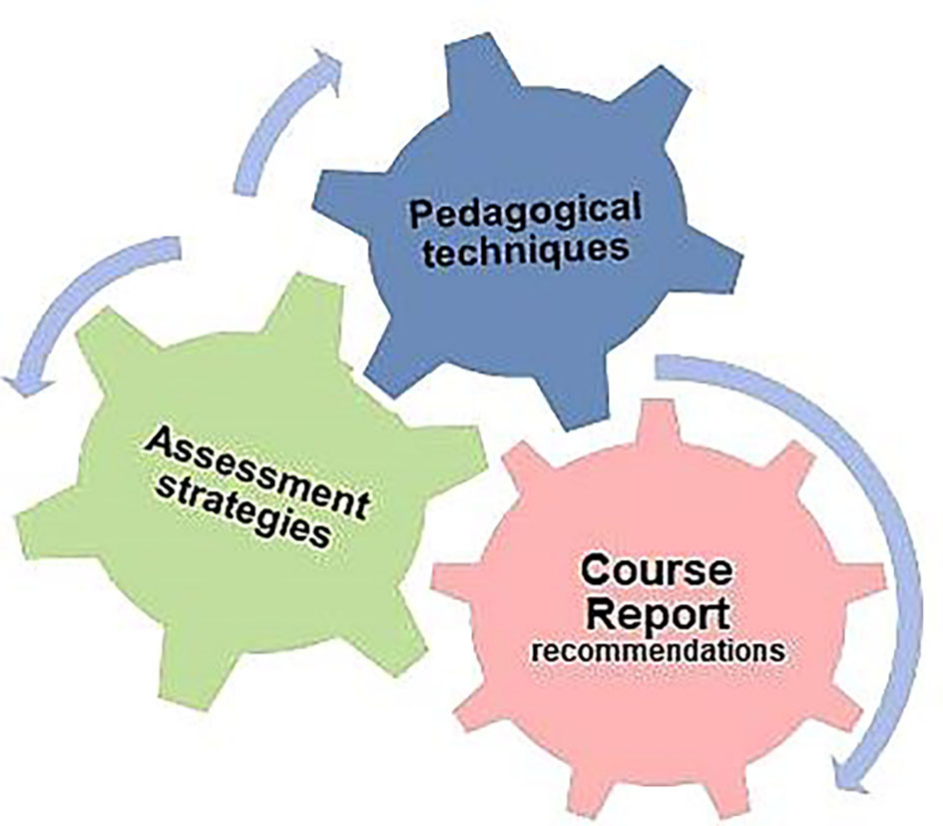
Course Reports recommendations; the main driver to modify learning/teaching strategies and assessment plans

- Course reports analysis supports the evaluation of the successful application of courses and conveys the dynamics of the whole programme comprehensively. These recommendations provided key improvements in the courses and served as the main driving force to upgrade the whole curriculum map as shown in figure 2. For instance, in the pre-clerkship phase (Year 1-4), we observed that few hard courses (Neurosciences, in level 6) were delivered before the basic body systems (Musculoskeletal course, in level 7). As a result, the student’s performance and scores in the neuroscience course were inadequate. Therefore, the course director prompted the directives in the curriculum map and consequences of this change were reflected positively in the subsequent years, particularly student performance in the formative and summative results. Student satisfaction was enhanced with this alteration as was effective curriculum mapping, which was imperative to coordinate the whole curriculum ( Harden, 2001).

- Student feedback by the end of each course was highlighted in the course reports. Their opinions and voice were supported by evidence. The analysis of their feedback helped to make amendments in the schedule, effective integration of content, timetables, teaching methods and assessments strategies. For example, learning in small groups and PBL discussions were perceived as more effective than lectures. This helped the course directors to work with course specifications to build more task-based, student-centred and teamwork activities. Moreover, they recognized the assessment tool which aligns with objectives and teaching plans, was based on this feedback.
- Using the analysis of student’s formative and summative assessments, content experts determined how to modify the course prerequisites and co-requisites. For instance, in the original curriculum plan, a pharmacology course was not a prerequisite for body system courses. Including the pharmacology course before the body systems courses improved student understanding, confidence and examination result both for pharmacology and the body systems courses. It also resulted in very positive feedback from the students.
- For quality assurance, the introduction of a quality unit helped to enhance the quality of courses. Their remarks provided a coherent and panoramic view of individual courses and programme on the whole.

### Evaluation Tools

- Self-evaluation by using self-study report could be used as a tool for OBE integrated curriculum. The application of the self-study report under the supervision of the quality unit helped to assess our progress in the programme itself and prompted to bridge the gaps in the applied curriculum. The self-reflection to enhance the OBE integrated curriculum was appreciated by faculty and students. Moreover administrative issues, for instance, logistics, resources, manpower, technology support; were highlighted by reviewers. These concerns were presented to stakeholders and refinement of shortcomings in the programme, were evaluated to enhance the delivery of the curriculum.
- Internal and external review by the Centre for Medical Education team from Hampton UK (CenMEDIC) helped to augment the quality of curriculum delivery ( Walsh, 2013). This aspect of evaluation prepared the institution for accreditation of the MBBS programme by governing authorities (SaudiMed), which included institutional recognition in the higher education sector.

### Constructive Alignment

- The course reports highlighted the percentage of achievements of each learning objective in three learning domains (cognition, psychomotor skills and attitude) assessed by different assessment tools (single best answers, very short answer questions, objective structured clinical skill exams, practical and logbook evaluation). This microscopic evaluation served as a corrective instrument to constructively align the individual course specifications, therefore this approach cultivated the OBE integrated curriculum by lining up the programme learning objectives, teaching strategies and assessment plans ( Essary and Statler, 2007; Harden, 2001).
- Moreover, modifying assessment strategies in the form of reflective practices in clinical courses and the introduction of team-based learning (TBL) was promoted. As a result, there was an introduction to the use of reflective journals in the clinical courses, especially in clinical and elective courses. Consequently, the reflective writing skills for learners were improved. Similarly, critical thinking stimulated deep learning among the students, in addition to identifying deficiencies and further learning goals ( Majid, 2016).

### Technology

- Currently, the cutting edge technological revolution in the educational environment, digitalization of learning management, and the introduction of 3D virtual reality (VR) have prompted an upgrade of the curriculum. The unique concept of telepresence and telemedicine will reshape future medical institutions. It will not only remodel the medical schools but also modernize the pedagogical techniques. The use of audience response systems (ARS), interactive lecturing, videos, different polling software, and artificial intelligence (AI) has created a stimulating and engaging learning environment. Therefore, the progression to adaptive and mastering learning ought to be encouraged, stimulated in the form of test-enhanced learning to uphold cognition & clinical skills ( Larsen, Butler and Roediger, 2008).
- Moreover, the high fidelity simulators have helped to expand clinical skills and mastering learning ( Issenberg *et al.*, 2005; Maran and Glavin, 2003). Hence, the curriculum featured advanced simulators and technology, which was encouraged by reviewers. Convincingly, these activities were duly supported by learning management portals, technology-enhanced multiple quizzes, blended learning, and audience response systems in the MBBS programme.

### Innovation

- With the passage of time, the OBE integrated curriculum is developing an innovative approach in order to enrich the quality standards of higher education. There was a great concern for developing collaboration among medical institutions nationally and internationally during the review process of three years. We have taken the initiative to develop partnerships with other medical institutions to help review, develop and implement our joint visions for improving medical education ( Cate, O. ten, 2007).
- Additionally, there is a trend to adopt the curriculum and the methods of delivery which promote learner curiosity. It is evident that curiosity endorses deeper learning and engagement in the learning process ( Schmitt, F. F. and Lahroodi, R., 2008).The aspect of dissonance in problem-based learning and well-structured cases scenarios enhance the learner to think critically and logically. Our reviewers suggested and agreed to promote curiosity in learning and teaching and to provide faculty development to train staff to promote curiosity in teaching ( Pluck, G., & Johnson, H., 2011).

### Variations

- There were some factors that had a significant impact on the applied courses within the curriculum, such as the number of part-time and adjunct faculty, a cohort of low achievers within the student body, a lack of trained faculty, and lack of resources. During a patch of unfavourable circumstances, it was decided that there must be an institutional policy to embrace such challenging situations. Such disruption must not affect the planned and applied curriculum. Therefore, it was argued to adapt change gradually and any disruption in the educational cycle and interference in curriculum change ought to be steady.

### Establish principles and nurturing the learning environment

- In order to ensure the applicability and sustainability of OBE integrated curriculum, the authors strongly recommended developing the policies and procedures for curriculum review at the institutional level. This was prescribed on the development of standard operating procedures (sops) for curriculum review. However, it remained a debatable issue that how often and how far the curriculum can be amended during its implementation. Moreover, how to define the major and minor changes in the curriculum needed further exploration.
- It was critically reasoned during the review process that when the system forces teachers to teach the test, we are narrowing down the learning to the core part of the subject to get through the exams. As a result, there is a stressful situation and actually, this is not helping learners much. We need to prepare students for lifelong learning, not just for the exams; which underpins the OBE integrated curriculum. Hence, there must be an exciting space and real value of self-directed learning and directed self-learning in the programme.
- There is intense demand for curriculum which instigates interest and boosts the intrinsic motivation in students, including autonomy support, adequate feedback, and emotional support ( Kusurkar *et al.*, 2012). The assessors supported and applied the idea of academic mentorship in order to strengthen the academic support system and enhance emotional intelligence ( Cherry *et al.*, 2014). There was special consideration given to students with low achievement and potential dropouts, by offering extra office hours and academic support. Moreover, in a few courses, there was a switch to task-based learning (TBL) to promote effective communication and time management skills in the learning environment by challenging the learners.
- In the health care profession, there is significant stress and burnout due to performance in complex and diverse situations because of multi-tasking and high stake decisions, especially in emergencies. This can lead to a lack of self-awareness, psychological discomfort, and cognition distress in medical professionals. Therefore, these conditions provoked the medical educators to engage with a curriculum, which guides the learner, to stay calm in a tough situation and promote the self-awareness ( Cherry *et al.*, 2014).The physiologic benefits of meditation programme in medical education have been proved very operative ( MacLaughlin *et al.*, 2011). Some authors advocate that it’s time to establish the medical curriculum by introducing a few courses which encourage mindfulness to nurture the healthy working environment ( Cuff, 2019; Elder *et al.*, 2007).
- In short, other authors have suggested fostering a learning environment, which is friendly, collaborative, flexible; because such an atmosphere will help the apprentices to explore their hidden potential ( Genn, 2001). As a result, it may generate innovations in the medical profession and cultivate the talent of budding physicians through the enhancement of emotional intelligence to investigate beyond the boundaries of the health profession.
- Finally, the authors suggested that institutions should create an enthusiastic, engaging, and passionate learning environment and enjoy the real pleasure of Diffendoofer Day; a charming story of a school where learners relish their learning in the unperturbed way ( Carey, 2011).

## Conclusion

This article addresses the main driving forces for curriculum management and recognises the leading influences for curriculum organisation in view of cutting edge developments in biomedical sciences; technology-enhanced learning; globalization of the health system, and the availability of various pedagogical techniques. It highlights the impact and consequences of decisions taken, in view of recommendations made by stakeholders of the curriculum. Course reports recommendations and evaluations serve a major role in proposing the modifications in the course specifications and curriculum map.

Defining major and minor changes in the curriculum remains a grey area. Moreover, how often to make the amendments in course specifications based on course reports recommendations remains a disputed question and needs the establishment of institutional guidelines. The authors presented a “CREATIVE” way forward which we have found invaluable, both for curriculum developers and for engaging with our stakeholders. This prompt spells out the key factors to consider while revisiting the OBE integrated curriculum and guides reviewers to look at the curriculum from various dimensions. These factors are: **C**ourse **R**eport recommendations, **E**valuation tools (internal and external), **A**lignment of pedagogical strategies with assessment, **T**echnology, **I**nnovation, **V**ariations in the learning environment, and **E**stablishment of institutional guidelines. Implementation of these fundamental elements allowed us the development of a curriculum that meets the standards for international accreditation and helped the institution to form a cohesive team of educationalists.

## Take Home Messages

• Appraisal and evaluation of an OBE integrated curriculum is very challenging in view of evidence-based practices, rapid reforms in pedagogical techniques and technology
• Curriculum review assists to identify the loopholes in the integration of basic and clinical sciences and helps to break the barriers for true assimilation of curriculum
• Course reports recommendations function as the main way to propose modifications in the course specifications and curriculum map
• Delineating major and minor changes and frequency of amendments in course specifications remains a questionable task
• We propose **“CREATIVE”** approach while reviewing and reforming the curriculum on the grounds of best practice evidence

## Notes On Contributors

This study was supported by the Research Unit, Alfarabi College of medicine Riyadh.

Dr Shazia Iqbal is working as Assistant Professor & Director of Medical Education at Alfarabi College of Medicine, Riyadh, Saudi Arabia. She assists in the development and review of OBE integrated curriculum at medical institutions with a special interest in pedagogical techniques and innovative educational technologies.

Dr Shahzad Ahmad is working as an associate professor at Fatima MemorialCollege of Medicine & DentistryLahore, Pakistan. He is keen to improve the learning environment by endorsing technology-enhanced learning and artificial intelligence in health professional education.

Professor David Taylor is a co-author for this article. He is Professor of Medical Education and Physiology at Gulf Medical University (GMU) in Ajman, UAE. David does research in pedagogic theory, higher education and educational theory. He is Director of Studies for the Master in Health Professions Education, offered jointly between Gulf Medical University (GMU) and Foundation for Advancement of International Medical Education and Research (FAIMER).

Jaye McIsaac is a Senior Fellow of the Higher Education Academy (SFHEA). She is an expert curriculum developer and academic quality adviser in higher education. Sheis working at the School of Education at Auckland University of Technology, in New Zealand.

Dr Turky H. Almigbal is assistant professor, family and community medicine department, College of Medicine, King Saud University, Riyadh. His has a keen interest in curriculum development and review.

## Declarations

The author has declared that there are no conflicts of interest.

## Ethics Statement

At Alfarabi College of Medicine, this research was approved from ethical approval members dated on 17.09.2019 CMC#01091719. The research was conducted from September, 2016, through September, 2019 in accordance with the Declaration of Helsinki.

## External Funding

This article has not had any External Funding
