# Supplementary material for: A CREATIVE approach to designing a contemporary medical curriculum
Source: MedEdPublish (2016). 2020 Mar 6;9:38. [Version 1] doi: 10.15694/mep.2020.000038.1 (PMC10697502; doi:10.15694/mep.2020.000038.1)
Supplement: Peer review history

# Reviewer report: Reviewer response for version 1

**DOI:** 10.21956/mep.20007.r30497

**Published:** 2020-04-19

**Authors:** Mohamed Al-Eraky

**Affiliations:** 1 University of Dammam

This review has been migrated. The reviewer awarded 2 stars out of 5 Thanks for sharing this interesting article. I was captured by the CREATIVE title. Authors may need to indicate a specific application/uses for their CREATIVE model, either in curriculum design, development, mapping, analysis, review or evaluation. The model needs to be discussed in view of other related frameworks, such as: SPICES1, Curriculum Navigator2, PRISM3 or the Stufflebeam’s CIPP4 Models. There was a brief reference to the SPICES model, but without correlation with the suggested one to build a strong argument for the need of a new model. The description of the current curriculum needs further elaboration. For instance, if the MBBS curriculum of Alfarabi College is ‘integrated’, to which extent on the integration ladder5? The Methodology was not clear to me. How the authors come up with their model? In the Discussion, many of the recommendations were generic. For instance, “fostering a learning environment, which is friendly, collaborative, flexible”. Authors needs to educate readers and scholars on when, how and what to expect when using their CREATIVE model in their context. I hope my feedback would help you to apply your model in real practice. Good luck! 1. Harden RM, Sowden S, Dunn WR. Educational strategies in curriculum development: the SPICES model. Med Educ [Internet]. 1984 Jul;18(4):284–97. Available from: http://doi.wiley.com/10.1111/j.1365-2923.1984.tb01024.x2. Al-Eraky MM. Curriculum Navigator: aspiring towards a comprehensive package for curriculum planning. Med Teach [Internet]. 2012;34(9):724–32. Available from: http://www.ncbi.nlm.nih.gov/pubmed/226463003. Bligh J, Prideaux D, Parsell G. PRISMS: New educational strategies for medical education. Med Educ. 2001;35(6):520–1. 4. Robinson B, Collit T, Calder J, Cipp T, Stufflebeam D, Public O, et al. The CIPP approach to evaluation. Context Input Process Prod approach to Eval. 2002;(May):1–4. 5. Harden RM. The integration ladder : a tool for curriculum planning and evaluation. Med Educ. 2000;34(7):551–7.

Reviewer Expertise:

NA

No decision status is available

# Reviewer report: Reviewer response for version 1

**DOI:** 10.21956/mep.20007.r30498

**Published:** 2020-03-18

**Authors:** Trevor Gibbs

**Affiliations:** 1 AMEE

This review has been migrated. The reviewer awarded 3 stars out of 5 Although I enjoyed reading this paper, I did find it very difficult to read and understand because of its lack of flow and logical approach. I was not sure initially whether the authors were suggesting the CREATIVE approach to all curricula review or that they had created these CREATIVE headings to review their curriculum. There were many points where the authors adopted a very superficial approach, mentioning particular areas of researched approaches but I was not sure they applied them to their curriculum or wanted to do them at a later date.The authors spoke strongly of an Outcomes-Based Curriculum , but I was unclear how the CREATIVE approach matched specific LOs- examples would have helped here. I think that the authors had not looked at the evaluation from the users of the system i.e. employers to evaluate the programme. Like my co-reviewer an overview of the curriculum would have been helpful.All is not lost however in this piece of research. An improvement in flow and clarity of the paper , using more specific examples, connecting change to findings from evaluation and exlporing more effectively the early and later outcomes ( the results) would all improve this paper and make it more useful for all curriculum developers.

Reviewer Expertise:

NA

No decision status is available

# Reviewer report: Reviewer response for version 1

**DOI:** 10.21956/mep.20007.r30499

**Published:** 2020-03-07

**Authors:** P Ravi Shankar

**Affiliations:** 1 American International Medical University

This review has been migrated. The reviewer awarded 4 stars out of 5 I enjoyed reading this manuscript about the CREATIVE approach to curriculum management and evaluation. Rapid developments in a number of areas underlines the importance of a dynamic and responsive curriculum. The authors have identified technological change, telepresence, telemedicine and the development of artificial intelligence as important challenges. They have described the process of curriculum review at the institution in detail. They refer to the basic sciences curriculum a number of times in the paper. Readers may find it helpful if an outline of the overall and the basic sciences curriculum is provided. The authors refer to a number of issues in the manuscript. The paper reads a bit disjointed in certain areas as diverse issues are addressed briefly. The authors have addressed a number of vital areas in the modern medical curriculum which can be of benefit to curriculum developers and educators. I was not able to find mention of a curriculum management system in the manuscript. I am also not sure about when the medical school was started. Inputs from alumni and from employers of graduates can also provide valuable inputs regarding the curriculum.

Reviewer Expertise:

NA

No decision status is available
